# Increasing the Storability of Fresh-Cut Green Beans by Using Chitosan as a Carrier for Tea Tree and Peppermint Essential Oils and Ascorbic Acid

**DOI:** 10.3390/plants11060783

**Published:** 2022-03-16

**Authors:** Karima F. Abdelgawad, Asmaa H. R. Awad, Marwa R. Ali, Richard A. Ludlow, Tong Chen, Mohamed M. El-Mogy

**Affiliations:** 1 Vegetable Crops Department, Faculty of Agriculture, Cairo University, Giza 12613, Egypt; karima.abdelgawad@agr.cu.edu.eg (K.F.A.); soma_h_97@yahoo.com (A.H.R.A.); 2 Food Science Department, Faculty of Agriculture, Cairo University, Giza 12613, Egypt; marwa3mrf@agr.cu.edu.eg; 3 School of Biosciences, Cardiff University, Sir Martin Evans Building, Cardiff CF10 3AX, UK; LudlowRA@cardiff.ac.uk; 4 Key Laboratory of Plant Resources, Institute of Botany, The Innovative Academy of Seed Design, Chinese Academy of Sciences, Beijing 100093, China; chentong@ibcas.ac.cn

**Keywords:** *Phaseolus vulgaris*, browning, antioxidant, shelf life, ready to eat

## Abstract

The quality of fresh-cut green beans deteriorates rapidly in storage, which contributes to increased food waste and lower perceived customer value. However, chitosan (Cs) and certain plant essential oils show promise in reducing postharvest quality loss during storage. Here, the effect of Cs and the combinations of Cs + tea tree oil (TTO), Cs +x peppermint oil (PMO), and Cs + ascorbic acid (AsA) on the quality of fresh-cut green bean pods (FC-GB) is studied over a 15-d storage period at 5 °C. All four FC-GB treatments reduced weight loss and maintained firmness during storage when compared to uncoated FC-GB. Furthermore, all treatments showed higher total chlorophyll content, AsA, total phenolic compounds, and total sugars compared to the control. The best treatment for reducing microbial growth was a combination of Cs + AsA. Additionally, the combination of Cs with TTO, PMO, or AsA showed a significant reduction in the browning index and increased the antioxidant capacity of FC-GB up to 15 d postharvest.

## 1. Introduction

Consumer demand for minimally processed fresh fruit and vegetables, such as washed, peeled, or trimmed produce, has increased globally in recent years [1]. However, these processes can cause damage to plant tissues and result in physiological changes that reduce quality, such as wilting, enzymatic browning, and a reduction in bioactive or sensory compounds [2]. With consumers becoming increasingly critical of synthetic food additives to either enhance shelf-life or product appearance, there is a demand for natural additives or treatments to mitigate these quality losses [3]. Fresh-cut fruit and vegetables often have a reduced shelf life due to their higher respiration rate, evaporation, and transpiration [4]. Due to the increased handling and processing steps, there are multiple potential sources of microbial contamination. Both favorable growth conditions and time for proliferation of these microbes occur through the following storage methods [5,6]. Indeed, this poses a significant public health risk and research is ongoing into how to best to manage and detect such outbreaks before they cause harm [7].

Green beans (*Phaseolus vulgaris* L.) are a popular and widely consumed legume, with 1,579,489 ha of land cropped in 2020 (FAO, 2020). As well as being valued for their culinary properties, green beans contain several health promoting compounds including minerals, vitamins, and dietary fibers [8]. Unlike many beans which are harvested at seed maturity and dried, green beans are picked earlier in development, and thus have a limited shelf life, even under refrigerated storage conditions [9]. The shelf life and quality of fresh-cut green beans decreases more quickly in storage when the beans are trimmed and prepared to be a ready-to-eat product [1]. Handling the beans can cause mechanical shocks which may result in cracks and bruising, and the trimming process itself causes mechanical injury to the tissues, which all elicit physiological and biochemical responses within the bean [10]. Any wounding to the epidermis releases nutrient-rich fluids which promote microbial growth, and the wound site itself facilitates microbial infiltration of the tissues, further reducing quality [11]. The biological responses within the beans can also cause them to become tough and fibrous prematurely, which results in lowered organoleptic quality and economic value [12].

Many essential oils from plants contain bioactive compounds, and some act as antibacterial and anti-fungal agents [13]. For example, tea tree oil (TTO) and peppermint oil (PMO) have promise as postharvest treatments to prolong the shelf life of minimally processed fruit and vegetables. TTO significantly reduces postharvest disease in a range of fresh produce, including strawberry [14] and lettuce [15]. Likewise, PMO has a protective affect against postharvest diseases and extends the shelf life of fresh fruit such as table grapes [16] and dragon fruit [17]. Both oils are considered nontoxic and furthermore have antioxidant and anticancer properties, according to the United States Food and Drug Administration [18]. Radical scavenging activity was detected in both PMO and TTO, at a concentration of 50 µLmL^1^, whereby PMO demonstrated 54% radical scavenging activity and TTO 63.56%.

Ascorbic acid (AsA), or vitamin C, is an essential micronutrient for humans, which protects against scurvy, contributes to normal immune function and iron uptake, and plays a role in the prevention of chronic diseases such as heart disease, diabetes, and cancer [19]. Additionally to the beneficial human health aspects of AsA, it has been shown to reduce enzymatic browning in fresh-cut fruit and vegetables [20]. Indeed, AsA is widely used as a non-toxic treatment to reduce some of the deleterious impacts of fruit and vegetable processing [21,22].

One of the most effective natural edible coating agents for extending shelf life of fruit and vegetables is chitosan (Cs). Chitosan is a linear polysaccharide, made by treating chitin with an alkaline substance, and has long-established antibacterial and antifungal properties [23]. Furthermore, studies have shown that Cs extends shelf life and preserves the quality of fresh fruit and vegetables such as fresh-cut red pepper [24], fresh-cut cucumber slices [2], and fresh-cut melon [25]. The beneficial effect of postharvest Cs treatment on unprocessed green beans has been reported previously [26,27]; however, to the best of our knowledge, the effect of Cs treatment on fresh-cut green bean shelf life and quality has not been studied before. Additionally, there is no previous work studying the effect of Cs as a carrier of TTO, PMO, or AsA on the shelf life or quality of fresh-cut green beans (FC-GB). Here, we aim to evaluate the synergistic effect of these compounds on the shelf life and quality of fresh-cut green beans stored for up to 15 d at 5 °C.

## 2. Materials and Methods

### 2.1. Preparation of Plant Material and Treatments

Green beans (*Phaseolus vulgaris* L., cv. Hama, Seminis Seed Company, this cultivar is highly resistant to bean common mosaic virus) free from defects, damage, and uniform in diameter and length (14 cm in length and 6 mm in diameter), were manually harvested from a local private farm (Giza governorate, latitude 29.9769353) and transferred to the laboratory within 2 h. The pods were prepared by trimming both ends of the pod with a sterile sharp knife. The beans were then immersed in four different treatment solutions for 5 min: (1) Chitosan (Cs) (0.5%), (2) Chitosan (0.5%) + Tea tree oil (TTO) (0.5%), (3) Chitosan (0.5%) + Peppermint oil (PMO) (0.5%), (4) Chitosan (0.5%) + Ascorbic acid (AsA) (0.5%), and (5) Control (untreated). 

### 2.2. Preparation of Chitosan Solutions

The chitosan coating solution was prepared according to Jiang et al. [28]. Cs was added to a dilute acetic acid solution (0.5%, *w*/*w*) to a concentration of 1.2 % (*w*/*w*), and to this, 30% glycerol and 5% Tween 80 were added. Essential oil solutions were then added to the Cs solution at a 1:1 ratio by weight. The mixture was stirred for 1 h at room temperature and the pH was adjusted using glacial acetic acid to pH 4.5. Three combinations were also prepared: Cs with AsA, Cs with TTO, and Cs with PMO. The first was prepared by adding AsA to the Cs solution at a concentration of 0.5% (*w*/*v*). Then, the two essential oil combinations were prepared by adding TTO/PMO at a 0.5% (*v*/*v*) concentration to the Cs solution, and additional Tween 80 was added as an emulsifier at 10% *v*/*v* of the essential oil. Mixtures were homogenized using a high-performance disperser (IKA T25-Digital Ultra Turrax, Staufen, Germany) at 13,500 rpm for 5 min at room temperature.

### 2.3. Storage Experiment 

After the fresh-cut green beans were treated, they were dried of surface moisture in a laminar airflow hood for 2 h. They were then packed in perforated polypropylene bags (each containing 250 g) and the bags were sealed. The bags were then stored for 15 d at 5 °C and 90% relative humidity, and sampling occurred every 3 d. Each treatment was performed in triplicate and the whole experiment was repeated twice. Control samples were treated identically.

### 2.4. Assessment of Weight Loss, Firmness, and Total Soluble Solids

To determine weight loss, beans were weighed after drying and at every sampling point, and the weight loss percentage was calculated with the following equation:

*Weight loss* (%) = (*Initial weight − Final weight/Initial weight*) × 100

Firmness was measured with a digital penetrometer (PCE-PTR 200, Jupiter, FL, USA) with a 6 mm diameter probe (range 0 to 1 kg). Total soluble solids (TSS) were determined with a digital refractometer and expressed as Brix (Model PR101, Atago Co., Ltd., Tokyo, Japan). 

### 2.5. Determination of Chlorophyll and AsA Content

Chlorophyll content was determined according to Strain and Svec [29], whereby 0.5 g of the sample was homogenized with 5 mL dimethyl formamide and kept in the dark in a refrigerator for 48 h. The absorbance was then measured at 647 and 663 nm with a spectrophotometer (model UV-2401 PC, Shimadzu, Milano, Italia). The chlorophyll content of the extract was determined using the following equation:

Chlorophyll content = 25.8 × A647 + 4.0 ± A663

where A647 and A663 are the absorbance at 647 and 663 nm, respectively. Chlorophyll content was subsequently expressed as mg/g of tissue.

The AsA content of FC-GB was determined using the titrimetric method with 2,6-dichlorophenol indophenol. Green bean tissue (10 g) was homogenized in 90 mL 3% oxalic acid, then filtered using Whatman filter paper to remove particulates. The resulting filtrate (25 mL) was then titrated with 2,6-dichlorophenol indophenol as per the Association of Official Agricultural Chemists’ methodology [30]. 

### 2.6. Total Phenolic Compounds and Total Sugar 

Total phenolic compounds (TPC) were assayed with Folin–Ciocalteu reagent as described by Singleton et al. [31], with slight modifications. Green bean tissue (5 g) was homogenized in 5 mL 80% methanol. The filtered solution was mixed with 2.5 mL of Folin-Ciocalteu reagent (diluted 10 fold with distilled water) and a further 2.5 mL of distilled water added. The mixture was incubated at 25 °C for 5 min and then 2 mL of aqueous sodium carbonate solution (7.5%, *w*/*v*) was added. The final solution was mixed and incubated in the dark at room temperature for 1 h. The absorption was measured at 765 nm using a spectrophotometer (UNICO S2100, Cole Parmer Instrument, Vernon Hills, IL, USA), and the results were calculated from a standard curve as gallic acid equivalent (GAE) milligrams per 100 mg of fresh fruit weight. Total sugar content was assessed with the Anthrone test, with absorbance measured spectrophotometrically at 630 nm. Glucose was used as a standard. In brief, 200 mg of fruit were extracted three times at 80 °C with ethanol (80%). After that, the solvents were evaporated from extract and concentrated extract re-dissolved in 2 mL distilled water. One milliliter of sample extracts was combined well with 1.5 mL of anthrone reagent (0.2% in H_2_SO_4_). A boiling water bath was used to heat the sample to a boil. The solution was cooled to room temperature prior to being measured for absorbance. The presence of total sugars is indicated by the formation of the blue-green complex.

### 2.7. Determination of Browning Index and Antioxidant Capacity

One gram of green bean tissue was homogenized in 10 mL ethanol (65% *v*/*v*) to determine the browning index according to Supapvanich et al. [32]. The mixture was stirred using a magnetic stirrer for 30 min at room temperature. The extract was filtered using Whatman No. 1 filter papers, then the absorbance was measured at 420 nm using a spectrophotometer (Unico UV-2000, UNICO company, Fairfield, NJ, USA). The result was calculated from the average of three absorbance replications. 

The antioxidant activity of the green bean tissue was determined using a modification of the method of Baardseth et al. [33]. One gram of green bean tissue was immersed in 10 mL of 80% methanol at room temperature and shaken for 30 min on a continually moving mechanical shaker. The extraction was carried out three times. Afterwards, the extracts were filtrated using Whatman No. 1 filter paper, then 0.1 mL of the extracts were diluted in 3.9 mL freshly prepared DPPH solution (2.4 mg DPPH in 100 mL 100% methanol). This mixture was then incubated in the dark at room temperature for 30 min. Absorbance was measured spectrophotometrically at 517 nm. The following equation was used to calculate the percent of radicals that was suppressed due to the antioxidant activity of green bean extracts:

% Inhibition = {(A_control_ − A_sample_)/A_control_} × 100

A_control_ = absorbance of the methanolic solution of DPPH only.

### 2.8. Determination of Mold and Yeast and Total Counts

Ten grams of tissue from each treatment was homogenized with 90 mL sterile saline water for 2 min by a stomacher (Stomacher BW-400P, Turelab, Shanghai, China). Mold and yeast (MY) and total counts were assessed on potato dextrose and plate count agar, after incubation at 37 °C for 48 h and 28 °C for 5 d, respectively [34]. The results were expressed as log10 of colony-forming units per gram sample (CFU g^−1^).

### 2.9. Statistical Analysis

Statistical analyses of the pooled data from the two experiments were performed with SPSS 18.0 version (SPSS Inc., Chicago, IL, USA). Normality distributions in each experiment were checked using the Shapiro–Wilk test. An analysis of variance (ANOVA) was performed for each experiment separately according to a completely randomized design. A combined analysis of variance was also performed from the mean data from each experiment, to create the means for the different statistical analysis methods. Bartlett’s test was used to test the homogeneity of variance between sample groups. Tukey’s honest significant difference test (*p* < 0.05) was used to examine differences among treatment means. The results are presented as average ± standard error. Pearson correlation was employed to examine the correlations among measured parameters.

## 3. Results 

### 3.1. Chitosan Based Treatments Minimize Weight Loss and Maintain Firmness throughout Storage

**Figure 1:**
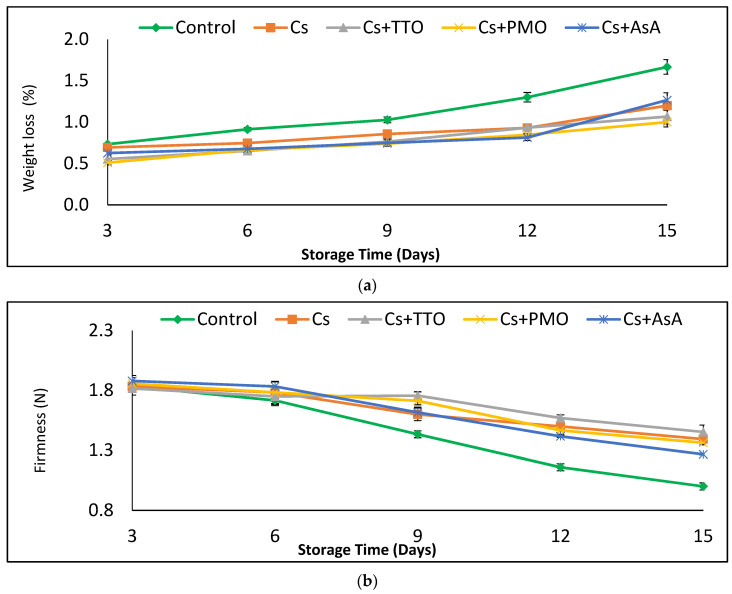
Effect of chitosan (Cs), Cs + tea tree oil (Cs + TTO), Cs + peppermint oil (Cs + PMO), and Cs + ascorbic acid (Cs + AsA) on (**a**) weight loss, and (**b**) firmness of fresh-cut green bean pods stored for 15 d at 5 °C. Error bars = SE, *n* = 6.

Weight loss in fresh-cut green beans was significantly reduced by all postharvest treatments from 6 d to 15 d of storage, compared to the control (Figure 1a; Table S1. Furthermore, significant reductions in weight loss were also observed at 3 d between the control samples and both Cs + PMO and Cs + TTO. Indeed, Cs + PMO and Cs + TTO showed significantly lower weight loss than beans treated with Cs or Cs + AsA at 15 d, suggesting particularly strong beneficial effects towards the end of shelf life. Conversely, the four treatments showed no significant differences in their efficacy at reducing weight loss at days 6, 9, and 12. 

Firmness was initially maintained in all treatment and control groups, with no significant differences between treatments nor any decreases from 3 d to 6 d (Figure 1b; Table S1). However, treatments of Cs + TTO and Cs + PMO resulted in significantly firmer beans than the control after 9 d of storage and indeed all treatments maintained firmness significantly better than the control after 12 or 15 d storage (Figure 1b; Table S1). 

### 3.2. Total Soluble Solids, Total Chlorophyll, AsA, and Total Phenolic Compounds

**Figure 2:**
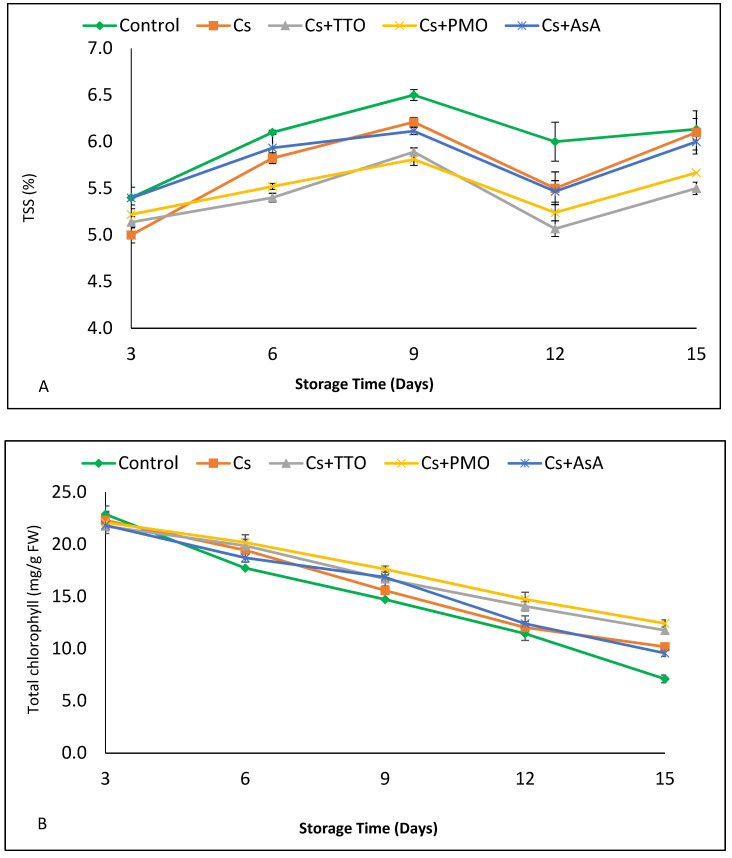

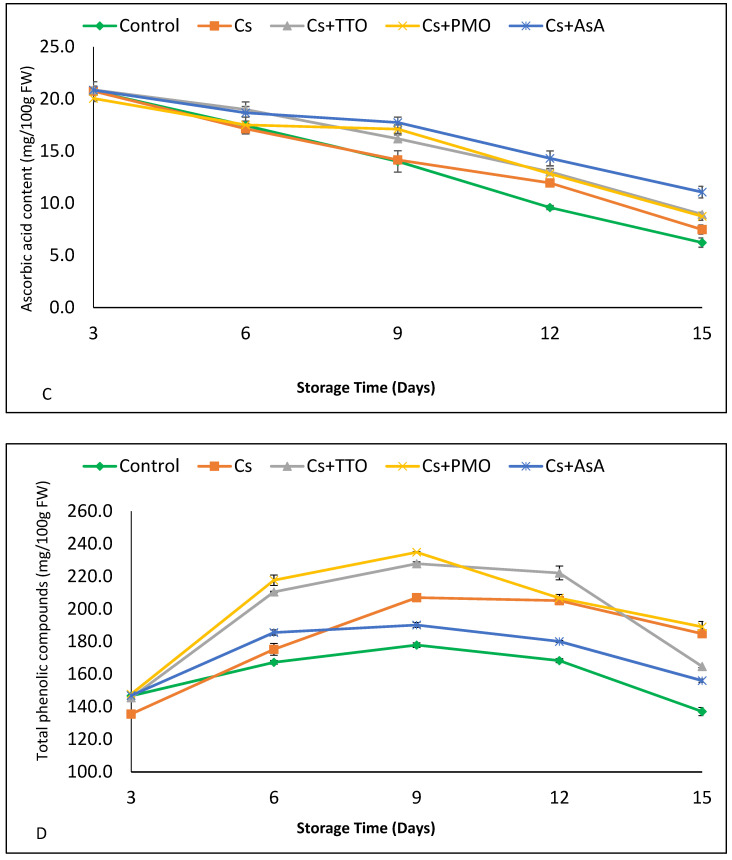
Effect of chitosan (Cs), Cs + tea tree oil (Cs + TTO), Cs + peppermint oil (Cs + PMO), and Cs + ascorbic acid (Cs + AsA) on (**A**) TSS, (**B**) total chlorophyll, (**C**) AsA, and (**D**) total phenolic compounds of fresh-cut green bean pods stored for 15 d at 5 °C. Error bars = SE, *n* = 6.

Total soluble solids in all samples of FC-GB significantly increased from 3 d to 9 d of storage and then all significantly decreased from 9 d to 12 d (Figure 2a). Control samples did not change significantly from 12 d to 15 d; however, all treatment groups showed a significant increase in TSS content over this time (Figure 2a). Within time points, the control samples typically had high TSS contents when compared to the treatment groups, and indeed at days 9 and 12, the control samples were significantly higher than all treatment groups. 

Total chlorophyll of FC-GB decreased over storage time in all treatment groups (Figure 2b). There was no difference between treatments after 3 d of storage; however, FC-GB treated with Cs + TTO and Cs + PMO had significantly higher total chlorophyll compared to the control and Cs + AsA at the end of storage. Indeed, Cs + PMO maintained total chlorophyll content at levels equivalent to values seen in the control sample three days earlier, between 6 d and 15 d of storage (Table S1). In addition, FC-GB treated with Cs alone and Cs + AsA had a higher chlorophyll content than the control after 15 d of storage. 

The AsA content of FC-GB decreased over storage time (Figure 2c). However, from 3 d to 6 d of storage, AsA levels did not fall by a significant amount in any treatment group. By day 9, Cs+ TTO, Cs + PMO, and Cs + AsA treatments maintained a significantly higher AsA content compared to the control and at 12 d and beyond, all treatments showed significantly higher AsA content compared to the control (Table S2). Whilst exogenous application of AsA in the Asa + Cs treatment group did not increase AsA content at the start of the experiment, this treatment resulted in the highest AsA content at 15 d, with significantly higher AsA concentrations than any other treatment or the control (Table S2). 

TPC initially increased and peaked at 9 d of storage before decreasing for the remainder of the storage period in all treatments and the control. TPC was higher in treated FC-GB than in control from 6 d until the end of storage. From 6 d to 12 d, the highest TPC concentration was found in Cs + TTO samples, and these were significantly higher than all other treatments (Figure 2d, Table S2). However, at 15 d, the concentration fell markedly in Cs + TTO, and Cs and Cs + PMO better maintained TPC concentration.

### 3.3. Total Sugar, Browning Index (BI), and Antioxidant Capacity

**Figure 3:**
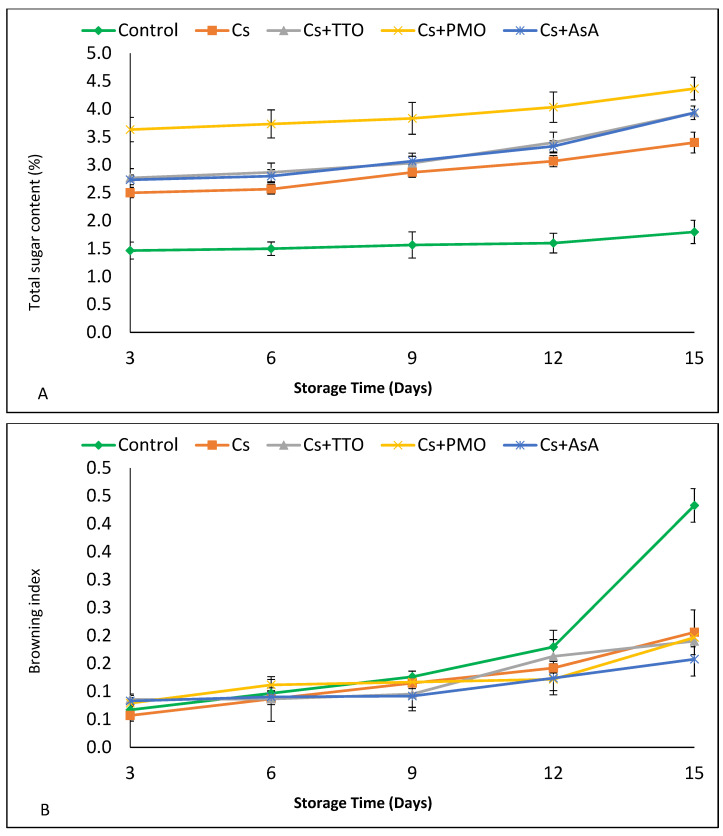

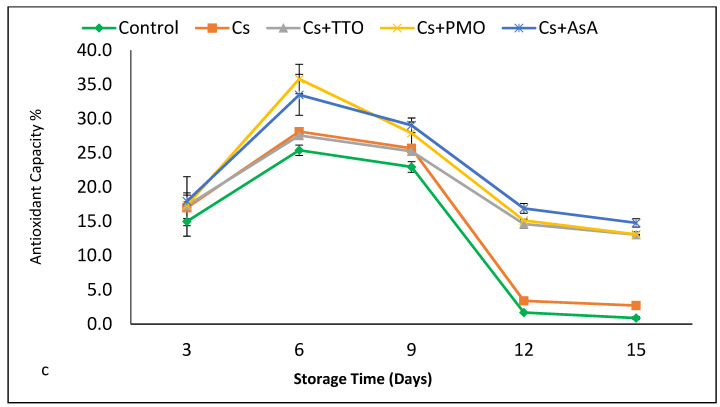
Effect of chitosan (Cs), Cs + tea tree oil (Cs + TTO), Cs + peppermint oil (Cs + PMO), and Cs + ascorbic acid (Cs + AsA) on (**A**) total sugar, (**B**) browning index, and (**C**) antioxidant capacity content of fresh-cut green bean pods stored for 15 d at 5 °C. Error bars = SE, *n* = 6.

The total sugar content of FC-GB was stable in the control group but increased over storage time in all treatments (Figure 3a). Throughout the time course, treated FC-GB had a higher total sugar content than the control (Figure 3a; Table S2). The highest sugar content was seen in Cs + PMO, where sugar contents were significantly higher than all other treatments at each time point (Figure 3a).

The browning index of FCGB was stable for the first 9 d of storage, with no treatments significantly changing from 3 d to 9 d (Figure 3b, Table S2). Browning significantly increased in Cs + TTO from 9 d to 12 d, but all other treatments and the control showed no significant differences (Figure 3b). From 12 d to 15 d, both the control and Cs + TTO significantly increased, and the control treatment showed the highest BI, with levels significantly higher than all other measurements (Figure 3b).

The antioxidant capacity increased significantly in all groups from 3 d to 6 d of storage then decreased after 9 d of storage (Figure 3c). At 12 d, Cs + TTO, Cs + PMO, and Cs + AsA treated samples lost significantly less antioxidant capacity than untreated samples or those treated with only Cs (Figure 3c; Table S2). Furthermore, at 15 d, the activity stabilized for all samples, and again Cs + TTO, Cs + PMO, and Cs + AsA treated samples displayed significantly higher activity than untreated or Cs (Figure 3c).

### 3.4. Mold and Yeast and Total Count

There were no colonies from mold and yeast or total counts detected in any sample at the first sampling point, at 3 d of storage. However, at 6 d and 9 d, colonies were present in both MY and total counts in the control samples and were not detected in any of the treated samples (Table 1). Furthermore, FC-GB treated with Cs + AsA suppressed both mold and yeast and total count until 12 d of storage, again with no colonies detected. FC-GB treated with either Cs + TTO and Cs + PMO had a significantly lower microbial load compared to the control at 12 d, and all treated beans had significantly lower microbial loads again at 15 d when compared to the control. Again, Cs + AsA had the lowest MY and total counts at 15 d, with significantly fewer colonies than all other treatments and the control, suggesting the combination of Cs + AsA offers the best antimicrobial properties of the treatments tested here.

## 4. Discussion

### 4.1. Appearance, Weight Loss, and Firmness

Appearance is an important quality aspect for consumers when deciding whether or not to purchase fresh produce. Previous studies indicate that the appearance of fresh stored shredded carrots is maintained by exogenous AsA application [35], which corroborates our results (Figure 1a). It has also been reported that AsA maintained quality by reducing chlorophyll degradation and delaying senescence in green bean pods [20]. Furthermore, El-Hamahmy et al. [36] found that exogenous application of Cs + AsA conserved quality and appearance of fresh stored pea pods, which have similar postharvest storage requirements to green beans. The ability of Cs to reduce weight loss and maintain phenolic compound levels and quality has been reported before [36]. Additionally, here we found strong negative correlation between appearance, weight loss, TPC, total count, mold and yeast, and browning (Table 2). Furthermore, appearance shows a positive correlation with chlorophyll and firmness. Exogenous application of TTO and PMO conserved the quality and appearance of strawberry and mangosteen fruit during cold storage, respectively [37,38], which is in agreement with our findings (Figure 1a).

Fresh fruit and vegetables lose weight after harvest due to transpiration and respiration [39] as well as structure of the cuticle [40]. The reduced weight loss of the FC-GB coated with Cs could be due to its ability to reduce transpiration and evaporation by forming a thin water-resistant film on the surface of fruit [41]. Additionally, the application of TTO, PMO, and AsA has been shown to reduce water loss during storage in strawberry, dragon fruit, and litchi fruit, respectively [17,37,42]. Our data suggest a modest synergistic effect of EOs and chitosan for reducing weight loss towards the end of shelf life, at 15 d. The reduction of weight loss following the application of chitosan and EOs could be due to their ability to decrease evaporation and transpiration rates [43]. The combination of chitosan and EO has been shown to have low water vapor permeability, linked to its hydrophobic nature [44,45,46].

FC-GB treated with Cs alone or combined with TTO, PMO, and AsA had higher firmness values compared with control (Figure 1b). In addition, the positive role of TTO and PMO for maintaining firmness of FC-GB might be due to their antimicrobial properties, thus reducing the degradation of pectin by spoilage microorganisms on the surface of FC-GB [47]. We report a correlation between an increase in microbial spoilage organism load and a loss in firmness in Table 2. Loss in firmness is predominantly driven by endogenous processes, such as the loss of turgor through evapotranspiration, and so it is possible that this correlation is not causal [48]. However, microorganism growth has also been shown to exacerbate losses in firmness [49]. Additionally, the strong positive correlation (r = 0.844, Table 2) seen between firmness and AsA content supports our result that firmness is enhanced by supplementary AsA application. This corroborates evidence in the literature, where sweet peppers treated with AsA maintained higher firmness [50].

### 4.2. Total Soluble Solids, Total Chlorophyll, AsA, and Total Phenolic Compounds

Cs coatings reduce the respiration rate of stored produce, and this has been shown to lower TSS in cherries by reducing the conversion of starch to sugar [51]. Conversely, however, higher TSS in control samples could arise due to greater weight loss in control samples from evaporation and transpiration, thus increasing solute concentrations within the bean. The positive correlation between TSS and weight loss in Table 2 may support this hypothesis.

Green color is the most important visual quality parameter that affects consumer’s acceptance of green vegetables such as green beans. Our results in Figure 2b supported our hypothesis that Cs coating delays chlorophyll degradation of FC-GB during refrigerated storage. This result agrees with Olawuyi et al. [52] who reported a reduction in yellowing of fresh-cut cucumber by Cs coating. There, the Cs coating reduced respiration rates, which resulted in lower rates of chlorophyll degradation, and it is possible that the same process also occurs in fresh-cut green beans, due to the similarity between these organs [52]. As well as Cs treatment, FC-GB treated with Cs plus TTO, PMO, or AsA showed higher chlorophyll content than the control. It was reported that TTO and PMO could protect plant tissues from oxidation and reduce the breakdown of chlorophyll pigments [53,54]. Additionally, AsA could reduce the loss of chlorophyll pigment by altering photosystem processes [55].

However, after the harvest of fresh fruit and vegetables, AsA concentrations rapidly decrease during processing and storage [39]. Our results shown in Figure 2c indicate that after 12 and 15 d of storage, all treatments maintained a higher content of AsA in FC-GB during storage compared to untreated FC-GB. The reduction of AsA loss in FC-GB coated with Cs may be due to its role in reducing O_2_ permeability. This was shown to lower the activity of the oxidative enzymes polyphenol oxidase (PPO) and peroxidase (POD), thus reducing the severity of enzymatic browning [56]. Furthermore, previous studies report the role of AsA treatment for maintaining AsA in litchi fruit [42]. A positive correlation was found between AsA in FC-GB and other measured parameters such as appearance, chlorophyll content, firmness, and antioxidant capacity (Table 2). While a negative correlation was recorded between AsA and other tested parameters (weight loss, TSS, mold and yeast, total count, and browning index).

Phenolic compounds are an important class of plant secondary metabolites, which have antioxidant properties [57]. Here, TPC content increased at the first three storage points and then decreased (Figure 2d), which mirrors the results obtained by Wang et al. [58] in loquat fruit. In this study, after 9, 12, and 15 d of storage, all treatments maintained the TPC of FC-GB better than the control. Similarly, previous work recorded a higher TPC in sweet cherry fruit coated with Cs [51]. In addition, TPC increased in litchi fruit in response to AsA application [42], and other study found that lettuce heads treated with TTO had higher TPC than the control [59]. TTO is hypothesized to regulate phenylalanine ammonium lyase activity, which is a key enzyme in the biosynthetic pathway of phenolic compounds, resulting in greater concentrations of TPC in the pods [59]. Likewise, an increased TPC was seen in white button mushrooms following PMO treatment [60]. As well as increasing TPC biosynthesis, EOs can also reduce the oxidation of existing phenolic compounds and delay the senescence process, further contributing to the overall increase in TPC [38].

### 4.3. Total Sugar, Browning Index, and Antioxidant Capacity

As shown in Figure 3a, FC-GB treated with Cs maintained total sugar concentrations that were higher when compared to the control, which is in accordance with findings in tomato, where fruit coated with Cs also contained higher total sugars [61]. The positive effect of either TTO or PMO in conserving total sugar has been attributed to the role of these EOs in decreasing the respiratory enzymes, resulting in lower sugar loss [62]. Furthermore, previous work observed higher total sugars in mushrooms treated after harvest with PMO compared to the control [60]. In addition, previous studies indicate that treatment with AsA and Cs+ AsA minimizes the reduction of total sugar of strawberry and litchi fruit during cold storage compared to the control [63,64].

A food product’s color, taste, and flavor are all affected by phenolic compounds and so are their health-promoting effects. Enzymatic browning is considered one of the most critical factors in the marketability and quality of fresh-cut vegetables, which is related to phenolic compounds [65]. Two enzymes are involved in the oxidative breakdown of phenolic compounds, which results in the formation of brown polymers, called melanines. The first of these enzymes is polyphenol oxidase (PPO; EC 1.14.18.1), which is a copper-containing enzyme found in many plants that can be either latent or active and performs a variety of reactions in the presence of oxygen and produces hydrogen peroxide. In the presence of hydrogen peroxide, a second enzyme called peroxidase (POD; EC 1.11.17) performs single electron oxidation on a broad range of substances, leading to browning. Therefore, PPO consider the promoter of POD activity due to its propensity for producing hydrogen peroxide during the oxidation of phenolic compounds [66]. Previous work indicated that exogenous postharvest application of AsA could decrease the browning of fresh-cut vegetables [39,67], which agrees with our findings (Figure 3b). The negative correlation between browning index and AsA in Table 2 further proves that exogenous application of AsA decreases the browning of FC-GB. This is likely linked to the antioxidant properties of AsA [67]. Chitosan is commonly used to reduce the browning of fresh produce during storage such as longan fruit [68] and loquat fruit [69]. The role of Cs in reducing browning index might be due to its role in reducing oxidative reaction in plant tissue [70]. Furthermore, pre-storage Cs + TTO application was shown to reduce browning and maintain quality in fresh-cut red bell peppers [24]. Our results shown in Figure 3b agree with previous study which found that browning of white mushrooms decreased by exogenous PMO treatment [71]. This could be due to the role of PMO in inducing antioxidants in tissues, which could delay browning [60]. Furthermore, the negative correlation between browning index and antioxidant capacity in Table 2 supports this hypothesis.

According to our results in Figure 3c, the antioxidant capacity in all treatments decreased over storage time, which is in agreement with previous study [72]. The reduction in antioxidant capacity during storage is due in part to the senescence of FC-GB resulting in the degradation of the cell structure and oxidation of its phenolic compounds [73]. Furthermore, our results agree with Xylia et al. [35] who reported that antioxidant capacity in fresh-cut lettuce was conserved by AsA, Cs, and essential oils (marjoram) treatment during cold storage. The ability of essential oils to increase antioxidant capacity in FC-GB could be a direct effect of their own high antioxidant activity [11]. However, it could also be in part due to their role in enhancing the activity of antioxidant enzymes, such as superoxide dismutase (SOD), catalase (CAT), and ascorbate peroxidase (APX) in plants [74,75].

### 4.4. Mold and Yeast (MY) and Total Count

Our results indicate that all treatments inhibited microbial growth until 9 d of storage (Table 1). Postharvest Cs application acts as an antibacterial agent against several postharvest diseases [76]. Additionally, several works demonstrated the inhibitive effect of EOs against human and plant pathogens [13,43]. Here, PMO, TTO, and AsA had an inhibitory effect on microbial growth on the surface of FC-GB. In previous studies, treated dragon and strawberry fruit with PMO and TTO inhibited the microbial growth during cold storage [17,37]. The inhibitory effect of EOs against the microbial growth is a product of its chemical composition, such as menthol and menthone found in PMO [77] and 4-terpinenol in TTO [78]. Menthol and menthone are shown to have potent antimicrobial properties against Gram positive and negative bacteria, yeast, and fungus. Likewise, 4-terpeineol has antibacterial properties [20,79]. Furthermore, our result may be related to the fact that AsA application reduces the pH, which can make conditions unfavorable for microbial growth [35]. Indeed, the negative correlation between AsA and mold and yeast in Table 2 supports our result that the postharvest application of AsA reduces the microbial growth on the surface of FC-GB.

## 5. Conclusions

Our study shows that Cs, and the combination of Cs+ TTO, Cs+ PMO, and Cs+ AsA can significantly prolong the shelf life of FC-GB by reducing weight loss and maintaining chlorophyll content, total soluble solids, firmness, AsA, total phenolic compounds, and total sugar. The combination of Cs+ AsA was the most effective treatment for reducing microbial growth during cold storage at 5 °C for up to 9 d. We propose that this treatment warrants further exploration as a novel tool for reducing postharvest losses in minimally processed vegetables and fruit.

## Figures and Tables

**Table 1:** Effect of chitosan (Cs), Cs + tea tree oil (TTO), Cs + peppermint oil (PMO), and Cs + ascorbic acid (AsA) on mold and yeast and total count (log CFU/g) of fresh-cut green bean pods stored at 5 °C for 15 d. Data are the average of 3 replicates ± standard errors. Different letters indicate significant differences (Tukey test, *p* < 0.05%).

	Mold and Yeast (CFU/g)				
	3 d	6 d	9 d	12 d	15 d
Cs	ND *	ND	ND	1.58 ± 0.02 a	1.68 ± 0.02 b
Cs + TTO	ND	ND	ND	1.37 ± 0.03 b	1.45 ± 0.01 c
Cs + PMO	ND	ND	ND	1.36 ± 0.01 b	1.43 ± 0.02 c
Cs + AsA	ND	ND	ND	ND	1.19 ± 0.01 d
Control	ND	1.45 ± 0.05 a	1.86 ± 0.03 a	1.68 ± 0.02 a	1.79 ± 0.03 a
	Total count (CFU/g)				
	3 d	6 d	9 d	12 d	15 d
Cs	ND	ND	ND	1.78 ± 0.02 a	1.70 ± 0.02 b
Cs + TTO	ND	ND	ND	1.53 ± 0.02 b	1.45 ± 0.01 c
Cs + PMO	ND	ND	ND	1.59 ± 0.08 b	1.61 ± 0.01 b
Cs + AsA	ND	ND	ND	ND	1.11 ± 0.02 d
Control	ND	1.61 ± 0.03 a	1.76 ± 0.03 a	1.73 ± 0.01 a	1.95 ± 0.02 a

* ND = not detected.

**Table 2:** Pearson’s correlation analysis between the physicochemical properties of fresh-cut green beans.

	Appearance	Weight Loss	Chlorophyll	AsA	TPC	Firmness	TSS	M & Y	Total Count	Sugar	Browning Index
Weight loss	−0.722 **										
Chlorophyll	0.811 **	−0.853 **									
AsA	0.867 **	−0.848 **	0.933 **								
TPC	−0.305 **	−0.164	−0.111	−0.098							
Firmness	0.688 **	−0.836 **	0.865 **	0.841 **	0.054						
TSS	−0.295 *	0.491 **	−0.422 **	−0.366 **	0.96	−0.372 **					
M & Y	−0.618 **	0.796 **	−0.769 **	−0.805 **	−0.77	−0.723 **	0.295 *				
Total count	−0.586 **	0.756 **	−0.762 **	−0.804 **	0.023	−0.698 **	0.369 **	0.919 **			
Total sugar	−0.180	−0.157	−0.154	−0.184	0.400 **	0.007	−0.297	−0.058	−0.034		
Browning	−0.735 **	0.821 **	−0.748 **	−0.784 **	−0.131	−0.754 **	0.296 **	0.626 **	0.571 **	−0.20	
Antioxidant	0.541 **	−0.642 **	0.575 **	0.619 **	0.376 **	0.627 **	0.053	−0.657 **	−0.584 **	0.079	−0.585 **

** Correlation is significant at the 0.01 level (2-tailed); * correlation is significant at the 0.05 level (2-tailed).

## Data Availability

The data presented in this study are available in article and Supplementary Material.

## Supplementary Materials

The following are available online at https://www.mdpi.com/article/10.3390/plants11060783/s1, Table S1: Effect of treatments on weight loss, firmness, TSS, and total chlorophyll, Table S2, Effect of treatments on vitamin C, total phenolic compounds, and total sugar.
