# Supplementary material for: Increasing the Storability of Fresh-Cut Green Beans by Using Chitosan as a Carrier for Tea Tree and Peppermint Essential Oils and Ascorbic Acid
Source: Plants (Basel). 2022 Mar 16;11(6):783. doi: 10.3390/plants11060783 (PMC8954194; doi:10.3390/plants11060783)
Supplement: Supplementary file 1 [file plants-11-00783-s001.zip › plants-1641207-supplementary.pdf]

Supplementary table S1: Effect of treatments on weight loss, firmness, TSS, and total chlorophyll

Appearance	3 days	6 days	9 days	12 days	15 days
Cs	5±0.10 a	4±0.10 b	4±0.10 a	3±0.10 b	2±0.20 c
Cs+TTO	5±0.10 a	4±0.10 b	3±0.20 b	3±0.10 b	3±0.10 b
Cs+PMO	5±0.10 a	4±0.20 b	4±0.10 a	4±0.20 a	3±0.10 b
Cs+AsA	5±0.10 a	5±0.10 a	4±0.20 a	4±0.10 a	4±0.20 a
Control	5±0.10 a	5±0.10 a	4±0.10 a	3±0.10 b	2±0.10 c
Weight loss	3 days	6 days	9 days	12 days	15 days
Cs	0.69±0.04 a	0.74±0.01 b	0.85±0.03 b	0.93±0.02 b	1.20±0.06 b
Cs+TTO	0.55±0.00 a	0.65±0.02 c	0.76±0.04 bc	0.93±0.01 b	1.06±0.09 b
Cs+PMO	0.51±0.03 a	0.66±0.01 bc	0.74±0.02 c	0.84±0.01 b	1.00±0.06 b
Cs+AsA	0.62±0.02 a	0.67±0.02 bc	0.75±0.04 c	0.81±0.04 b	1.26±0.09 b
Control	0.73±0.01 a	0.913±0.02 a	1.02±0.04 a	1.30±0.06 a	1.66±0.09 a
Firmness					
Cs	1.83±0.07 a	1.78±0.04 a	1.60±0.03 ab	1.50±0.03 a	1.39±0.03 a
Cs+TTO	1.81±0.07 a	1.75±0.05 a	1.75±0.05 a	1.57±0.02 a	1.45±0.03 a
Cs+PMO	1.85±0.03 a	1.78±0.07 a	1.71±0.03 a	1.46±0.03 a	1.36±0.06 a
Cs+AsA	1.88±0.02 a	1.83±0.09 a	1.61±0.03 a	1.41±0.03 a	1.26±0.02 a
Control	1.83±0.04 a	1.71±0.04 a	1.43±0.04 b	1.16±0.01 b	1.00±0.01 b
TSS					
Cs	5.00±0.00 b	5.82±0.02 ab	6.21±0.06 b	5.50±0.21 ab	6.10±0.12 a
Cs+TTO	5.13±0.09 ab	5.40±0.06 c	5.89±0.05 cd	5.06±0.18 b	5.50±0.23 b
Cs+PMO	5.22±0.06 ab	5.52±0.05 bc	5.81±0.04 d	5.24±0.08 ab	5.66±0.07 ab
Cs+AsA	5.39±0.10 a	5.93±0.03 a	6.11±0.06 bc	5.46±0.09 ab	6.00±0.00 ab
Control	5.40±0.12 a	6.10±0.15 a	6.50±0.04 a	6.00±0.12 a	6.13±0.09 a
Total chlorophyll					
Cs	22.31±0.81 a	19.44±0.03 ab	15.58±0.08 b	12.04±0.65 ab	10.19±0.36 b
Cs+TTO	21.74±0.77 a	19.87±0.26 ab	16.70±0.24 ab	14.07±0.57 a	11.77±0.34 a
Cs+PMO	22.11±0.43 a	20.18±0.61 a	17.62±0.69 a	14.74±0.44 a	12.42±0.32 a
Cs+AsA	21.83±1.07 a	18.71±0.76 ab	16.85±0.29 ab	12.40±0.67 ab	9.57±0.35 b
Control	22.88±0.41 a	17.73±0.41 b	14.72±0.23 c	11.45±0.75 b	7.11±0.32 c

Supplementary table S2: Effect of treatments on vitamin C, total phenolic compounds, and total sugar.

Vitamin C	3 days	6 days	9 days	12 days	15 days
Cs	20.75±0.90 a	17.15±0.80 b	14.15±1.02 bc	11.95±0.19 c	7.47±0.45 d
Cs+TTO	20.88±0.22 a	18.99±0.44 b	16.19±0.12 c	13.01±0.16 d	8.93±0.44 e
Cs+PMO	20.05±0.31 a	17.51±0.29 b	17.11±0.37 b	12.82±0.31c	8.75±0.14 d
Cs+AsA	20.85±0.30 a	18.67±0.36 b	17.74±0.40 b	14.30±0.12 c	11.06±0.38 d
Control	20.75±0.39 a	17.46±1.04 b	14.01±0.52 c	9.58±0.72 d	6.23±0.56 e
TPC					
Cs	135.50±1.61 d	175.23±1.18 c	206.94±1.39 a	205.15±1.17 a	184.80±2.55 b
Cs+TTO	145.55±2.21 d	210.46±3.65 b	227.77±1.35 a	222.09±0.22 a	164.74±1.38 c
Cs+PMO	147.75±1.36 e	217.63±0.39 b	234.81±1.18 a	206.65±4.42 c	189.06±0.59 d
Cs+AsA	146.86±0.98 b	185.56±3.17 a	190.18±0.39 a	180.07±2.31 a	156.04±3.34 b
Control	146.68±0.77 c	167.27±1.61 b	177.86±1.54 a	168.30±0.59 b	137.05±0.97 d
Total suagr					
Cs	2.50±0.15 b	2.56±0.12 ab	2.86±0.23 ab	3.06±0.18 ab	3.40±0.21 a
Cs+TTO	2.76±0.09 c	2.86±0.09 bc	3.03±0.09 bc	3.40±0.10 ab	3.93±0.19 a
Cs+PMO	3.63±0.17 b	3.73±0.17 b	3.83±0.18 ba	4.03±0.19 a	4.36±0.12 a
Cs+AsA	2.73±0.22 b	2.80±0.25 ab	3.06±0.28 ab	3.33±0.27 ab	3.93±0.20 a
Control	1.46±0.09 b	1.50±0.12 b	1.56±0.09 b	1.60±0.10 b	1.80±0.06 a
Browning index					
Cs	0.06±0.01 b	0.09±0.01 b	0.11±0.02 a	0.14±0.03 ab	0.21±0.03 b
Cs+TTO	0.09±0.01 a	0.09±0.01 b	0.10±0.01 b	0.16±0.02 ab	0.19±0.04 b
Cs+PMO	0.08±0.01 a	0.11±0.04 a	0.12±0.03 a	0.12±0.03 b	0.20±0.01 b
Cs+AsA	0.08±0.01 a	0.09±0.01 b	0.09±0.02 b	0.12±0.02 b	0.16±0.01 b
Control	0.07±0.01 ab	0.10±0.01 ab	0.12±0.01 a	0.17±0.03 a	0.43±0.03 a
Antioxidant capacity					
Cs	17.01±3.20 a	28.13±0.99 abc	25.68±0.25 ab	3.40±1.00 c	2.72±0.95 c
Cs+TTO	17.30±0.85 a	27.56±2.70 bc	25.25±2.80 ab	14.63±0.30 b	13.06±0.67 a
Cs+PMO	17.36±2.20 a	35.81±3.10 a	27.90±1.80 a	15.15±1.26 ab	13.11±1.00 ab
Cs+AsA	17.95±3.74 a	33.49±1.30 ab	29.04±1.34 a	16.90±0.15 a	14.79±0.30 b
Control	15.00±3.20 a	25.40±0.71 c	22.96±0.65 b	1.70±0.67 c	0.89±0.56 d
